# Ex vivo delivery of recombinant IL-10 to human donor lungs

**DOI:** 10.1016/j.jhlto.2024.100192

**Published:** 2024-12-06

**Authors:** Jonathan C. Yeung, Terumoto Koike, Dirk Wagnetz, Tiago N. Machuca, Riccardo Bonato, Mingyao Liu, Stephen Juvet, Marcelo Cypel, Shaf Keshavjee

**Affiliations:** Latner Thoracic Surgery Laboratories, University Health Network, Toronto, Ontario, Canada

**Keywords:** lung transplantation, ex vivo lung perfusion, interleukin-10, drug delivery, aerosol mechanics

## Abstract

**Background:**

The immunoregulatory cytokine interleukin-10 (IL-10) has been shown to be a promising therapy for donor lung injuries before transplantation. However, the very short half-life of IL-10 in vivo (∼2 hours) has necessitated the use of gene therapy in almost all animal models of lung transplantation. Because isolation of the donor lung on the ex vivo lung perfusion (EVLP) circuit removes it from the influence of renal and hepatic clearance mechanisms, a much-prolonged half-life of IL-10 is anticipated. Thus, we hypothesized that delivery of recombinant IL-10 (rIL-10) to injured donor lungs isolated on EVLP could be a clinically relevant and a logistically simpler method of employing IL-10 therapy in lung transplantation.

**Methods:**

Injured human donor lungs clinically rejected for transplantation were split into single lungs and the better of the 2 subjected to 12 hours of EVLP and randomized (*n* = 5/group) to receive either saline (control), rIL-10 (5 µg in 2-liter perfusate), or rIL-10 (25 µg) aerosolized into the airways.

**Results:**

Perfusate and intratracheal delivery of rIL-10 did not provide the therapeutic anti-inflammatory action that has been traditionally achieved with gene therapy. It appears that intratracheally delivered rIL-10 moves into the perfusate where it seems to be biologically inactive.

**Conclusions:**

Gene therapy remains superior as it allows for continued production of IL-10 within the alveoli where it has the potential to continuously act on alveolar macrophages and epithelial cells in a paracrine fashion.

## Background

Normothermic ex vivo lung perfusion (EVLP) preserves the metabolic activity of the lung during preservation and allows for the deployment of ex vivo lung repair strategies.^1^, ^2^ Our laboratory has had success with utilizing interleukin-10 (IL-10) in both small and large animal models of lung transplantation to reduce ischemia-reperfusion injury.^3^, ^4^, ^5^ IL-10 is a cytokine that has been shown to inhibit the production of proinflammatory cytokines and chemokines (i.e., IL-1α, IL-1β, IL-6, IL-12, IL-18, granulocyte-macrophage colony-stimulating factor (GM-CSF), granulocyte colony-stimulating factor (G-CSF), macrophage colony-stimulating factor (M-CSF), tumor necrosis factor (TNF), leukemia inhibitory factor (LIF), and platelet activating factor (PAF)) by macrophages and neutrophils, in vitro.^6^ Indeed, the alveolar macrophage has been shown to play a major role in the initiation of ischemia-reperfusion injury^7^ and IL-10 can regulate the amount of proinflammatory cytokine production by these cells.^8^ But because the half-life of IL-10 in vivo is short (around 2 hours),^9^ use of recombinant IL-10 (rIL-10) would require almost continuous delivery to the donor. This would be logistically challenging for a multiorgan donor and may cause unexpected or undesirable effects on other donor organs. Hence, most preclinical therapeutic strategies delivering IL-10 to the lung have employed targeted gene therapy, cell therapy, or clustered regularly interspaced short palindromic repeats (CRISPR)-based delivery strategies in the ex vivo setting to avoid the aforementioned logistical and off-target effects.^5^, ^10^, ^11^, ^12^, ^13^, ^14^

Our group has been studying IL-10 therapy using EVLP. When IL-10 was delivered via an intratracheal (IT) adenoviral-mediated IL-10 transgene to clinically declined human lungs during EVLP, beneficial effects included reduced proinflammatory cytokine formation and improved lung function with a diminished vector-associated inflammatory response.^15^, ^16^ We have subsequently developed strategies for IL-10 transcriptional upregulation using CRISPR in vitro, rat lung transplant, and human lungs on EVLP.^5^, ^17^

Nonetheless, during the development of EVLP and IL-10 gene therapy, it became apparent that the IL-10 protein does not breakdown as readily during ex vivo perfusion and demonstrates a much-prolonged half-life, likely due to the absence of renal and other plasma-based cytokine clearance mechanisms, such as proteolytic degradation and circulating soluble IL-10 receptor in the plasma. We thus hypothesized that the use of rIL-10 could be a simpler method of EVLP IL-10 delivery and anticipated that strategies developed for the delivery of rIL-10 could be generalized to other potential protein and small molecule ex vivo organ therapeutics.

In this study, we examined the delivery of rIL-10 by IT and intravascular routes to clinically declined human donor lungs on the normothermic EVLP circuit. Furthermore, we assessed the effectiveness of rIL-10 delivery during EVLP in improving human lungs for transplantation.

## Materials and methods

### Design

Human lungs clinically declined for transplantation were placed on EVLP and then randomly assigned to receive intravenous (IV) rIL-10 (*n* = 5), IT rIL-10 (*n* = 5), or saline vehicle (*n* = 5). EVLP was continued for 12 hours after delivery.

### Human lungs

Injured human lungs clinically declined for transplantation by all lung transplant programs with donor consent for research were utilized. These donor lungs were recovered with the intention of clinical use and were declined by the transplant team at the time of intraoperative donor evaluation. The better of the 2 lungs were utilized to maximize the potential of completing 12 hours of EVLP and none of the contralateral lungs were used for clinical transplant. Institutional research ethics board and Trillium Gift of Life Network research approval were also obtained.

### Ex vivo lung perfusion

Single human lung EVLP was performed using the Toronto EVLP technique as detailed by Cypel et al for 12 hours.^1^ Compliance and airway pressure measurements were recorded from the ventilator (Servo-I, Maquet, Wayne, NJ). PaO_2_ and PvO_2_ were measured with an arterial blood gas monitor (RapidLAB 348, Siemens, Deerfield, IL). Standardized lung inflation was performed by measuring lung parameters exactly 10 minutes following recruitment of the lung to a pressure of 25 cm H_2_O. Evaluation occurred at an FiO_2_ of 21% and 100%.

## Delivery of recombinant IL-10

### Intratracheal aerosolization

Carrier-free recombinant human IL-10 (25 μg, R&D Systems) was dissolved in 6 ml of phosphate-buffered saline. An Aeroneb Go vibrating membrane aerosol generator was attached to the ventilator on the inhalational arm of the ventilator. The endotracheal tube was cut to 15 cm and the tip of the tube was placed 2 cm proximal to the first bronchial bifurcation. This was confirmed by fiberoptic bronchoscopy. The lung was fully recruited and then the ventilator was set to a tidal volume of 6 ml/kg (for a single lung) and a respiratory rate of 15 breaths per minute with a hold of 2 seconds for aerosol delivery. Aerosol was continuously generated during the time of delivery. This usually lasted 30 minutes for delivery of 6 ml of aerosolized solution. Tidal volumes were returned to standard EVLP ventilation parameters (3 cc/kg for a single lung) afterward.

### Intravascular delivery

Carrier-free recombinant human IL-10 (5 μg, R&D Systems) was dissolved in 2 ml of Steen solution (XVIVO Perfusion, Denver, CO) and added directly to the priming volume of Steen solution in the perfusion circuit of EVLP.

### Biopsies

Biopsies of the superficial portions of the lung were performed at 1 hour, at 3 hours, and then at every subsequent 3 hours using a GIA60 (Medtronic, Mansfield, MA) stapler. To assess the distribution of IL-10 delivered, 3 biopsies per lobe were taken at various depths of the lung at the end of perfusion. Perfusate samples were also taken with each biopsy.

### Inflammatory profile in human lung tissue biopsies

Lung tissue homogenization and protein extraction were performed as previously described.^4^ Human IL-8, IL-1β, IL-6, IL-10, TNF-α, and IL-12p40 were measured by flow cytometry in lung tissue homogenates using a fluorescent cytometric bead array assay according to the manufacturer's instructions (Human Inflammation Kit; BD Biosciences, San Jose, CA). Each human inflammation capture bead suspension (10 µl/test) was mixed. Fifty µl of the mixed capture beads were subsequently added to assay tubes containing 50 µl of the Human Inflammation PE Detection Reagent and 50 µl of sample or standards. The mixture was incubated for 3 hours and washed. Finally, the bead pellet was suspended and analyzed on a flow cytometer (LSR II, Becton Dickinson Immunocytometric Systems, San Diego, CA) using BD CellQuest Software. For formatting sample data and subsequent analyses, the BD CBA Software was used.

### Statistics

All results were expressed as mean ± standard error of the mean. For comparisons between the 2 groups at all timepoints, 2-way analysis of variance (ANOVA) was utilized. For comparisons between 3 or more groups, 1-way ANOVA was utilized. Post-test analysis between each group was performed with Bonferroni correction for multiple comparisons. *p*-values less than 0.05 were considered significant.

## Results

Median age, cold ischemic time until EVLP, last PaO_2_ in the donor, and reason for rejection did not differ between groups (Table 1).

**Table 1:** Characteristics of Injured Human Donor Lungs

	Control	IV IL-10	IT IL-10	Significance
Median age in years (range)	42 (25-58)	58 (13-81)	24 (15-39)	*p* = 0.26
Cold ischemic time until EVLP in hours (range)	5 (4-10)	6 (4-11)	5 (5-6)	*p* = 0.50
Last PaO_2_ in donor hospital in mm Hg (range)	290 (80-365)	303.5 (233-439)	423 (270-435)	*p* = 0.31
Reason for rejection (number)	Pneumonia (4), PA hypertension (1)	Pneumonia (4), donor malignancy (1)	Pneumonia (3), aspiration (1), emphysema (1)	

Abbreviations: EVLP, ex vivo lung perfusion; IL, interleukin; IT, intratracheal; IV, intravenous; PA, pulmonary artery.

### Recombinant IL-10 delivered ex vivo is measurable 12 hours after delivery in tissue and perfusate

**Figure 1:**
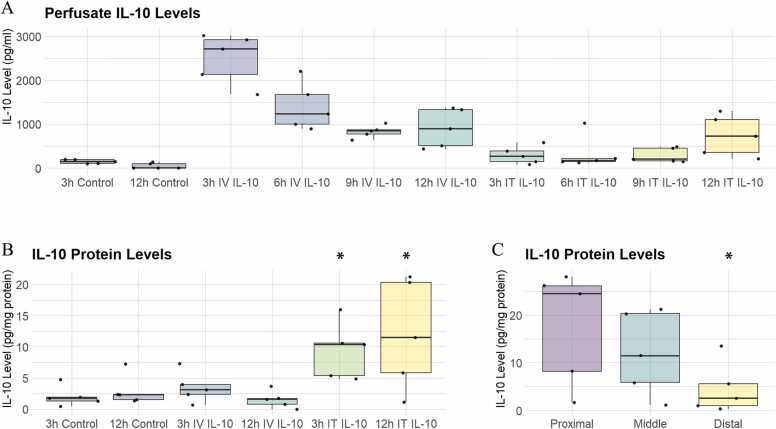
(A) IL-10 levels in perfusate by delivery method (x axis). (B) IL-10 levels in lung tissue by delivery method (x axis). (C) IL-10 levels in lung tissue at proximal airways, middle airways, and distal lung in intratracheally delivered rIL-10. *Indicates *p* < 0.05. Data shown as median and interquartile range. IL, interleukin; IT, intratracheal; IV, intravenous.

Following the delivery of 5 μg of rIL-10 into 2 liter of perfusate, we expected to achieve approximately 2,500 pg/ml of IL-10. Indeed, we measured 2,493 pg/ml ± 255.5 after 3 hours of perfusion. By 12 hours of perfusion, the level of IL-10 in the perfusate fell to 908.8 ± 196.6 pg/ml. This corresponds to a half-life of 8.23 hours. In contrast, IL-10 perfusate levels in the control group remained essentially absent at both timepoints. For the IT-delivered group, IL-10 appeared to gradually leak out into the perfusate as IL-10 perfusate levels increased to 293.7 ± 88.8 pg/ml at 3 hours of perfusion then increased to 738 ± 208.8 pg/ml by 12 hours of perfusion (Figure 1A).

When we examined IL-10 levels in lung tissue, IT–delivered IL-10 levels were elevated compared to both control and the IV–delivered IL-10 group at 7.6 ± 2.6 pg/mg protein at 3 hours and 12.0 ± 3.9 pg/mg protein at 12 hours, *p* = 0.04. There were no significant differences between the IV rIL-10 group and the control group (Figure 1B).

### Distribution of IL-10 within the lung following IT delivery

To assess the distribution of rIL-10 within the lung following aerosolized delivery, we next examined the tissue distribution of IL-10 at the end of 12 hours of perfusion in the IT IL-10 group by taking biopsies along the airway and out into the periphery of the lung. Unsurprisingly, levels of IL-10 were higher in the tissue near the proximal airway than in the distal alveoli, *p* < 0.05 (Figure 1C).

### Effect of IL-10 on ex vivo lung physiologic parameters

We next examined the effect of IL-10 on the physiology of ex vivo perfused lungs. The IV IL-10 group had 1 lung which developed major edema during perfusion leading to an early termination of EVLP with corresponding falls in compliance and pO_2_ and increases in airway pressure. However, overall, there were no differences between the groups in pO_2_ at the end of EVLP.

**Figure 2:**
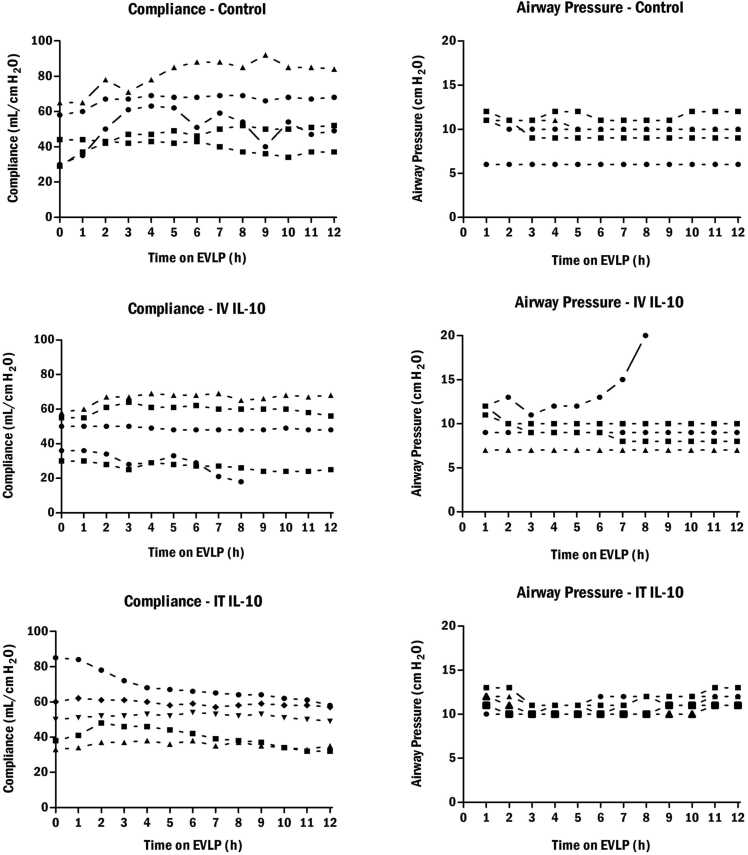
Compliance and airway pressure in ventilated lungs over 12 hours of EVLP (control: top; IV rIL-10: middle, and IT rIL-10 bottom). EVLP, ex vivo lung perfusion; IL, interleukin; IT, intratracheal; IV, intravenous; rIL, recombinant IL.

We next examined the physiologic parameters of compliance and airway pressure. Apart from the aforementioned lung in the IV IL-10 group, there were no significant differences between the compliances or airway pressures in the control, IV rIL-10 group, and the IT rIL-10 group. In the control group, compliance rose or was stable in all 5 cases. Compliance rose or was stable in 4 cases of the IV rIL-10 and this was mirrored in the IT rIL-10 group. A similar stability was found in airway pressure measurements between the 3 groups (Figure 2).

### Effect of rIL-10 on lung cytokine expression

**Figure 3:**
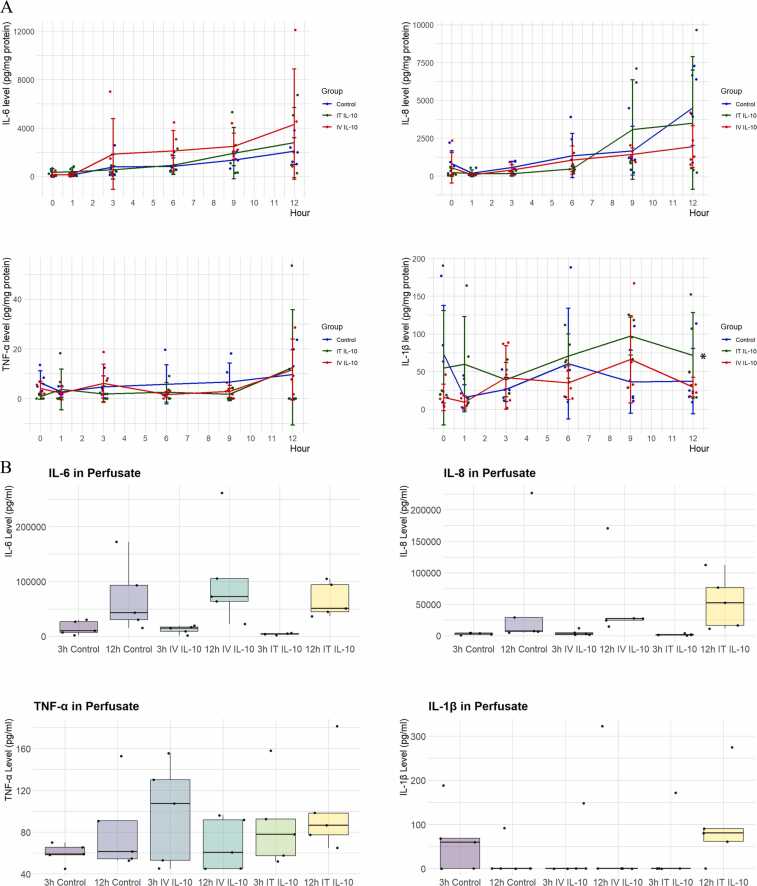
Cytokine levels (IL-6, IL-8, TNF-α, an IL-1β) in lung tissue following control, IV rIL-10, and IT rIL-10. *Indicates *p* < 0.05 compared to control. Data shown as median and interquartile range. IL, interleukin; IT, intratracheal; IV, intravenous; rIL, recombinant IL.

Most of the lungs had excellent pO_2_ and stable or improving compliances and airway pressures on EVLP. We examined the effect of rIL-10 on the expression of proinflammatory cytokines. Significant increases in tissue levels of IL-6 and IL-8 occurred in all groups, regardless of the route of rIL-10 administration. TNF-α levels remained mostly stable in all 3 groups. There was a significant elevation in IL-1β in the IT rIL-10 group compared to the other groups (*p* < 0.05 at 12 hours, *p* = 0.04 overall), whereas the other cytokines remained statistically similar (Figure 3A).

To avoid unnecessary injury to the lung, tissue biopsies during perfusion are taken from the periphery of the lung. These biopsies are assumed to represent cytokine production in the whole lung.^18^ Because cytokines are not cleared rapidly during EVLP, cytokines in the perfusate can be considered to represent the cumulative amount of cytokines produced by the entire lung. We therefore subsequently measured cytokine perfusate levels at 2 different timepoints. There was no difference in levels at 12 hours between the 3 groups. While the overall trend was similar to the tissue levels, the difference in IL-1β seen in tissue did not occur in the cytokine levels within the perfusate (Figure 3B).

## Discussion

The development of EVLP has given us the potential to effect meaningful change before implantation, during the time donor lungs spend outside of the body. Because it was anticipated that rIL-10 would have a prolonged half-life during EVLP, we assessed the effect of rIL-10 delivery during EVLP on donor lung function and inflammation during EVLP. rIL-10 was delivered to the lungs by 2 routes: intravascular and IT. For the intravascular route, we simply added rIL-10 to the circulating perfusate, whereas for the IT route, we utilized an aerosol delivery method to homogeneously deliver rIL-10 to the distal portions of the lung.

While aerosolized drug delivery is commonplace for the treatment of asthma and other proximal airway diseases, the delivery of aerosols deep into the lung parenchyma is more challenging. The cross-sectional surface area of the lung increases drastically with each successive branching of the airways. Consequently, there is a significant drop off in the velocity of flow as air moves distally into the lung. This has implications for aerosol delivery. Aerosol particles depend on a high flow velocity to push them around corners and into the distal lung. When flow decreases, larger aerosol particles, which possess more inertia, will fail to turn with the flow and impact onto the airway more proximally than smaller particles. While smaller particles are more likely to reach the distal portions of the lung, they also carry much less drug per particle since their volume is proportional to the cube of their radius. Hence, delivery time will be much longer for fine aerosols. In general, aerosol particles with a diameter of 3 to 5 µm will deposit in the central portion of the lung and particles with a diameter of 3 µm or smaller can be delivered more distally.

In the present study, we utilized an Aerogen Aeroneb Solo, a nebulizer system intended for ventilated patients. It generates an average aerosolized particle size of approximately 3.3 µm which balances delivery time with distal aerosol delivery. Delivery of an aerosol during EVLP has some potential benefits. First, humidified air is not needed for ventilation, thus moisture from the air is less likely to add to aerosol particle size during delivery. Second, removal of the lungs from the patient enables the use of ventilation strategies aimed at maximizing aerosol delivery without needing to consider the adequate ventilation of the patient, nor the cardiac consequences of aggressive ventilation. In this study, we achieved delivery of rIL-10 to the distal portions of the lung at a concentration of about 7 μg/mg protein. Indeed, as expected, proximal IL-10 levels were higher at around 15 μg/mg protein after 12 hours of perfusion.

When we measured the effect of rIL-10 either on measures of lung function or cytokine expression, we could not detect any differences from the control group. This is in stark contrast to a similar study performed by Cypel et al in which measures of lung function improved and tissue proinflammatory cytokine levels fell significantly following IL-10 delivery to the lung using IT adenoviral gene therapy.^15^ In that study, Cypel et al could demonstrate tissue IL-10 levels of around 30 pg/mg total protein from peripheral biopsies following gene therapy. This is higher than the level we could achieve with IT aerosol delivery. Interestingly, IV delivery of rIL-10 did not result in a measurable increase in tissue levels of IL-10, suggesting that IV rIL-10 could not enter the lung parenchyma.

The anatomical location of IL-10 delivery appears to be important to its biological function. The in vitro and in vivo effects of IL-10 center on reducing activation of antigen-presenting cells (APC). Indeed, APCs within the lung, such as the alveolar macrophage and dendritic cells, all express IL-10 receptor, and the alveolar macrophage plays a central role in cytokine expression and production following ischemia-reperfusion injury and other diseases.^19^, ^20^, ^21^ As an organ exposed to the outside environment, APCs within the lung, such as the alveolar macrophage, are present on the epithelial side of the barrier rather than the endothelial side. Thus, IL-10 may exert most of its beneficial effect on the epithelial side and this may explain the observation that IV–delivered rIL-10 appeared to be ineffective. This is only one potential explanation as IL-10 signaling is complex and the observed ineffectiveness may be due to other reasons, such as downstream signaling pathways.^22^

Alveolar delivery of rIL-10 is already difficult to achieve in high concentrations for reasons described above. Compounding this difficulty is the discovery that perfusate levels of rIL-10 were elevated by 3 hours of perfusion following IT delivery, suggesting that alveolar rIL-10 is cleared into the circulation over time and thus rIL-10 delivered IT only has a short time to act on APCs in the lung. IL-10 clearance from the lung is well-described. In studies from this laboratory and others studying IT adenovirus–mediated gene therapy, IL-10 levels increased in the circulation following IT delivery.^23^ Interestingly, in a study by Minter et al, a comparison was made between IT adenovirus–mediated therapy with human IL-10 and viral IL-10 and they found that viral IL-10 accumulated in the tissue at significantly increased levels compared to human IL-10.^24^ A future study comparing the hIL-10 to vIL-10 amino acid sequence may allow for the engineering of an IL-10 protein which prefers the tissue compartment.

The gene therapy approach may yet overcome these difficulties. Even small amounts of viral delivery to the alveoli will eventually result in large amounts of alveolar IL-10 as IL-10 will be produced by the alveolar cells themselves. This eliminates the difficulties associated with delivering large amounts of rIL-10 to the distal parenchyma. The other major problem is that IT rIL-10 appears to be rapidly cleared to the perfusate. Thus, rIL-10 delivered IT into the lung has only a short window of time to act on alveolar macrophages before leaking into the perfusate. Indeed, we were not able to achieve the same concentration of IL-10 in the peripheral lung and this may have been a reason for lack of effect. Since IL-10 delivered by gene therapy continuously produces IL-10 within the lung tissue, transgene IL-10 which leaks into the perfusate is continuously replenished. Continuous IT rIL-10 aerosolization during EVLP could be considered as an alternative solution; however, the difficulties of distal delivery of rIL-10 and potential airway obstructions from mucus or pneumonia remain.

In this study, we chose to deliver rIL-10 to clinically declined human donor lungs. Advantages to the use of these lungs include their human origin and their representation of the complexities of real-world multifactorial donor lung injury. However, there can be pitfalls in the utilization of these lungs for experimental studies. The Toronto Lung Transplant Program is an experienced high-volume program and utilizes a high percentage of donor organs. Moreover, this study was performed in parallel with a clinical trial studying EVLP evaluation of marginal donor lungs. Thus, the threshold for rejection for clinical use was much higher and resulted in the majority of the lungs utilized in this study being infected. In this respect, rIL-10 may not have been able to overcome this type of relatively extreme injury and that may have affected the limited observed benefit of rIL-10, particularly since pneumonia may cause pus to obstruct the distal airways with IT delivery. In using the “better” of the 2 lungs, however, we were able to complete 12 hours of EVLP without significant edema formation which implies that these lungs may actually have been clinically usable and may not have been as injured as initially thought. Indeed, we have since clinically utilized more of these “better” single lungs when the contralateral lung is declined.^25^ Nonetheless, while adenoviral gene therapy mediated IL-10 upregulation showed significant important anti-inflammatory and lung functional benefits in our previous work, the donor lungs studied were from a different era of lung transplantation and the injury may not be equivalent to the lungs studied here, which had more pneumonia as reason for decline.^15^ Indeed, the IT route of adenoviral vector delivery used in the previous study would similarly be blocked by pus or mucus in the airways of infected lungs.

Although rIL-10 delivery IT or IV did not appreciably affect lung function or cytokine production in this population of lungs, this study still demonstrated the concept of drug delivery during EVLP and the pharmacokinetics of rIL-10 delivery. The majority of drugs today are small molecules, rather than proteins, and thus may not be amenable to gene therapy strategies. Putative therapies using these drugs, such as antibiotics or β-adrenergic agonists, could benefit from aerosolized delivery to the lung and this study will aid in the development of these drug delivery strategies. In the context of clinical IL-10 therapy, rIL-10 delivery does not appear to be the most attractive option.

## Author contribution

Conceived and designed the analysis: J.C.Y., T.K., D.W., and S.K. Collected the data: J.C.Y., T.K., D.W., T.N.M., and R.B. Performed the analysis: J.C.Y., M.L., S.J., M.C., and S.K. Wrote the paper: J.C.Y., M.L., S.J., M.C., and S.K.

## Disclosure statement

Shaf Keshavjee reports financial support was provided by the Canadian Institutes of Health Research. Marcelo Cypel and Shaf Keshavjee report a relationship with Traferox that includes equity or stocks. M.C. and S.K. are consultants for Lung Bioengineering. There other authors declare that they have no known competing financial interests or personal relationships that could have appeared to influence the work reported in this paper.

Acknowledgments: None.

This work was supported by the Canadian Institutes for Health Research (#312227).
